# Supplementary material for: De Novo Design of Anti-COVID Drugs Using Machine Learning-Based Equivariant Diffusion Model Targeting the Spike Protein
Source: Curr Issues Mol Biol. 2023 May 12;45(5):4261–84. doi: 10.3390/cimb45050271 (PMC10217495; doi:10.3390/cimb45050271)
Supplement: Supplementary file 1 [file cimb-45-00271-s001.zip › Supplementary files/Supplementary file S3.pdf]

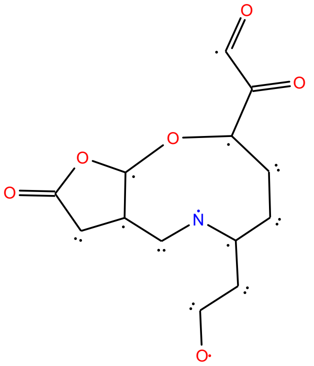

entry name spike_inpaint_ca_mol.1

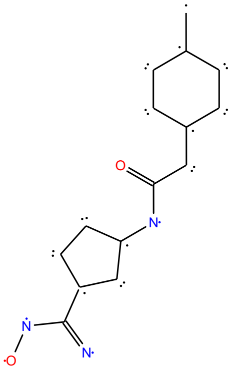

entry name spike_inpaint_ca_mol.2

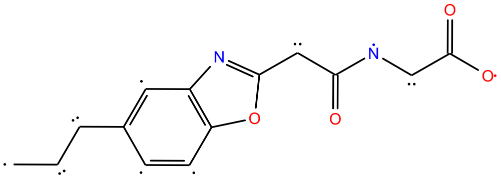

entry name spike_inpaint_ca_mol.3

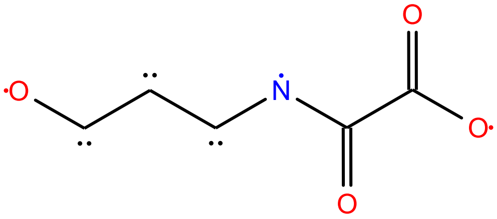

entry name spike_inpaint_ca_mol.4

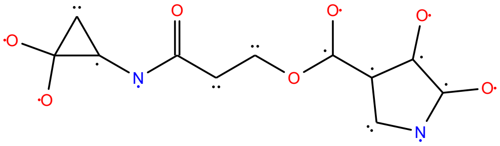

entry name spike_inpaint_ca_mol.5

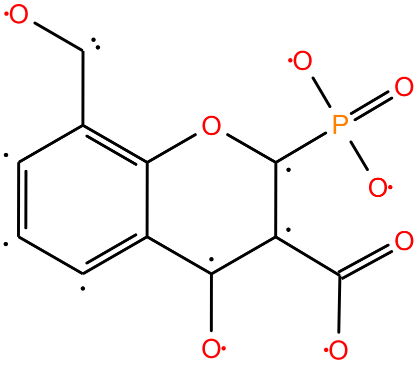

entry name spike_inpaint_ca_mol.6

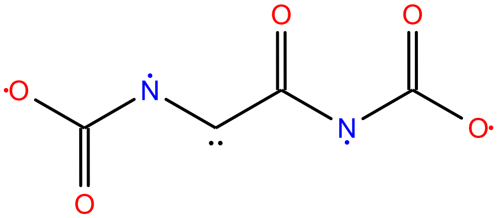

entry name spike_inpaint_ca_mol.7

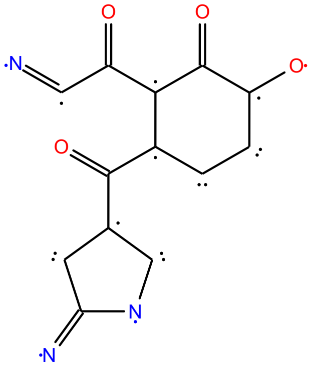

entry name spike_inpaint_ca_mol.8

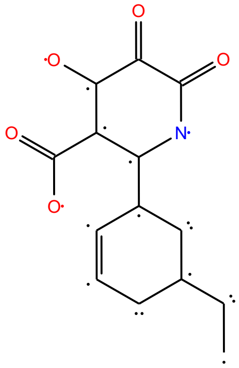

entry name spike_inpaint_ca_mol.9

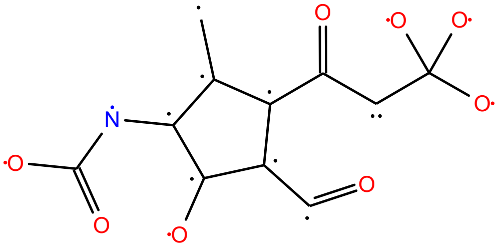

entry name spike_inpaint_ca_mol.1[illegible]

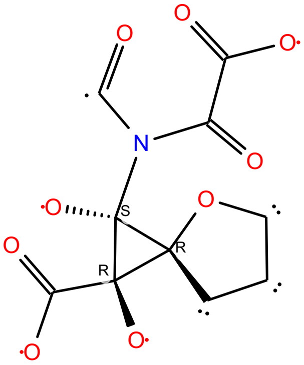

entry name spike_inpaint_ca_mol.1[illegible]

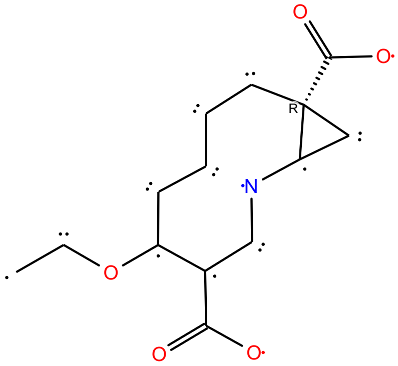

entry name spike_inpaint_ca_mol.1[illegible]

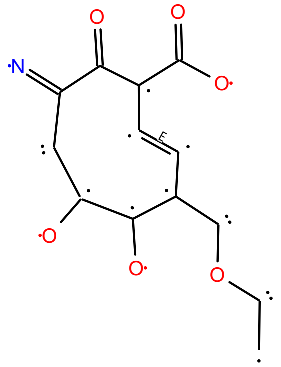

entry name spike_inpaint_ca_mol.13

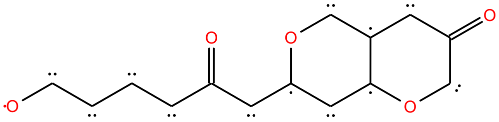

entry name spike_inpaint_ca_mol.14

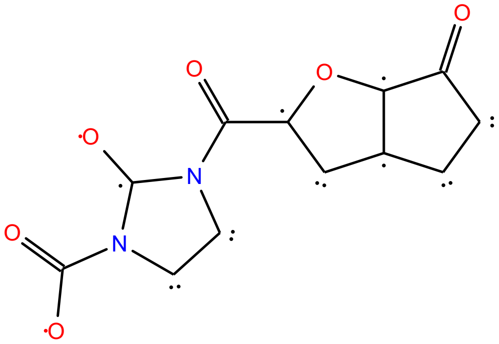

entry name spike_inpaint_ca_mol.15

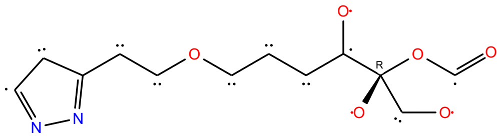

entry name spike_inpaint_ca_mol.16

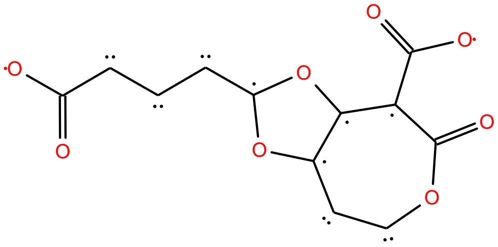

entry name spike_inpaint_ca_mol.17

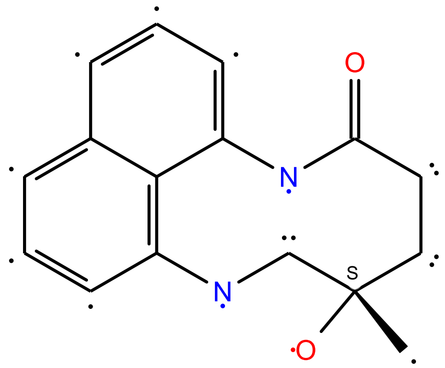

entry name spike_inpaint_ca_mol.18

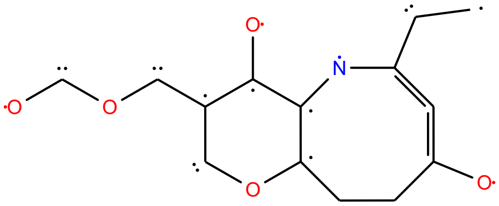

entry name spike_inpaint_ca_mol.19

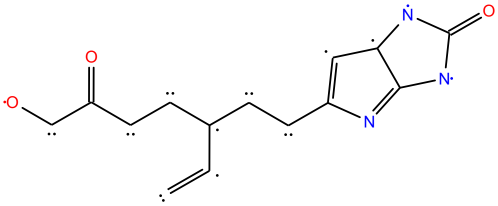

entry name spike_inpaint_ca_mol.20

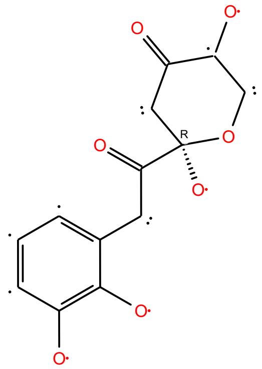

entry name spike_inpaint_ca_mol.21

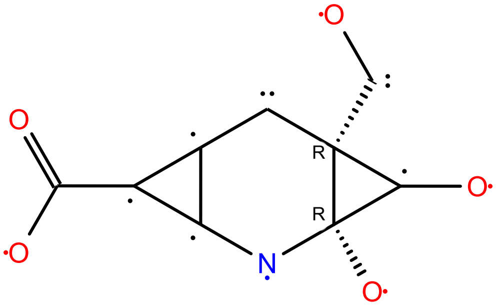

entry name spike_inpaint_ca_mol.22

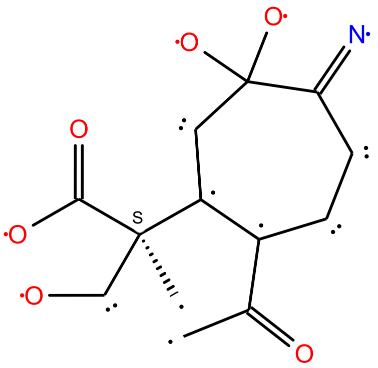

entry name spike_inpaint_ca_mol.23

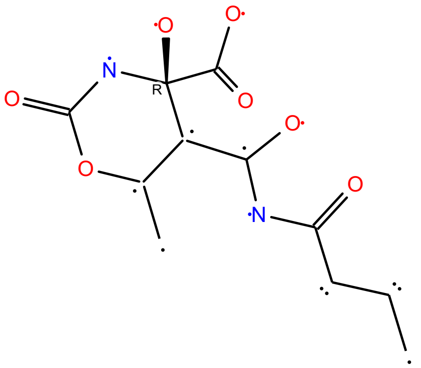

entry name spike_inpaint_ca_mol.24

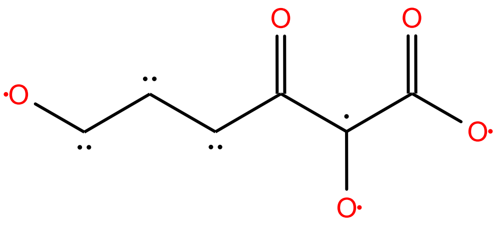

entry name spike_inpaint_ca_mol.25

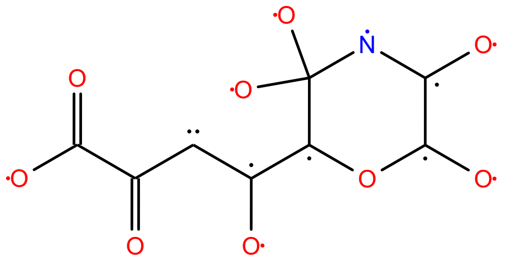

entry name spike_inpaint_ca_mol.26

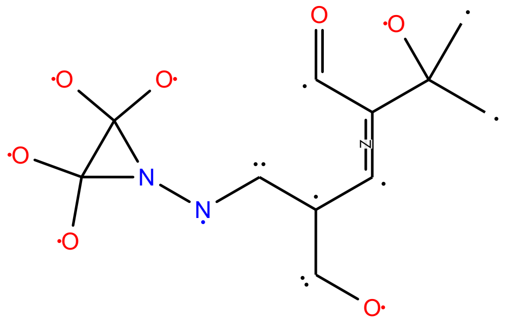

entry name spike_inpaint_ca_mol.27

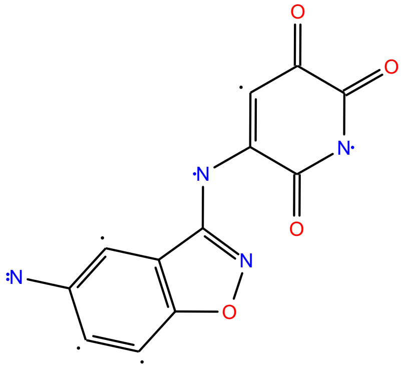

entry name spike_inpaint_ca_mol.28

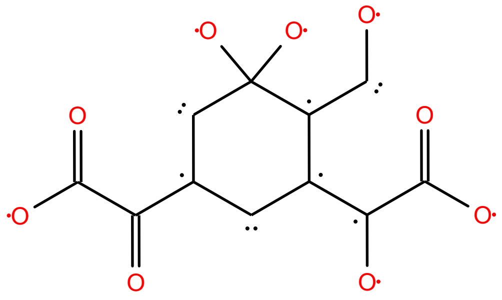

entry name spike_inpaint_ca_mol.29

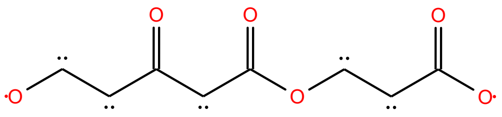

entry name spike_inpaint_ca_mol.30

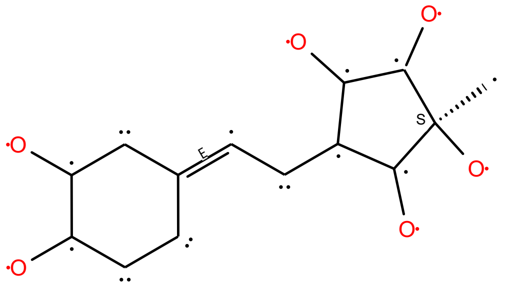

entry name spike_inpaint_ca_mol.31

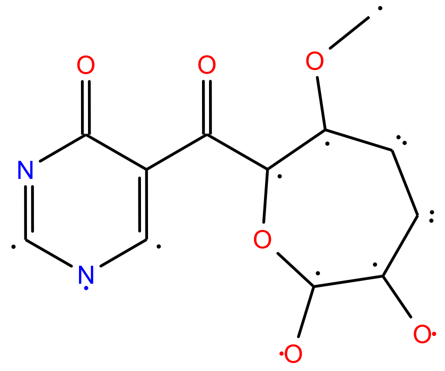

entry name spike_inpaint_ca_mol.32

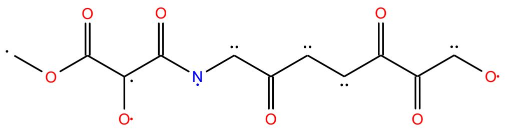

entry name spike_inpaint_ca_mol.33

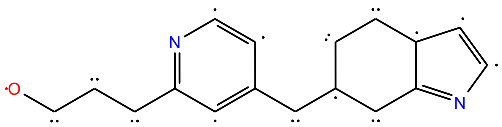

entry name spike_inpaint_ca_mol.34

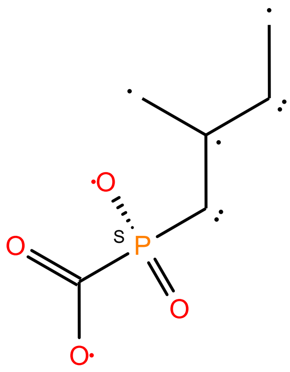

entry name spike_inpaint_ca_mol.35

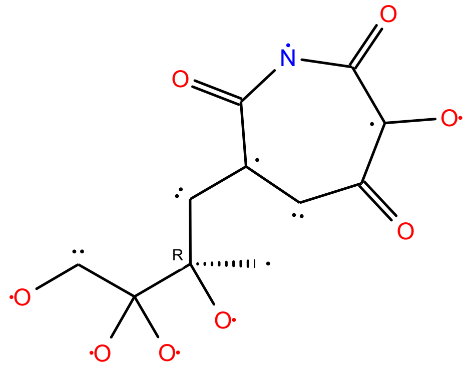

entry name spike_inpaint_ca_mol.36

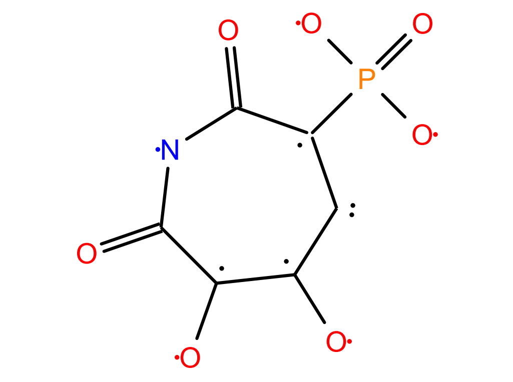

entry name spike_inpaint_ca_mol.37

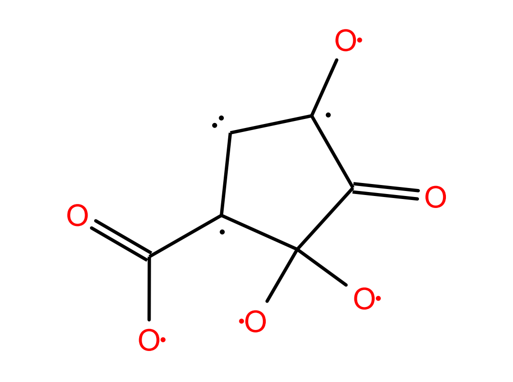

entry name spike_inpaint_ca_mol.38

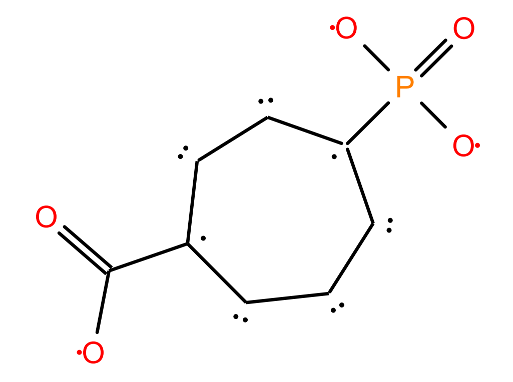

entry name spike_inpaint_ca_mol.39

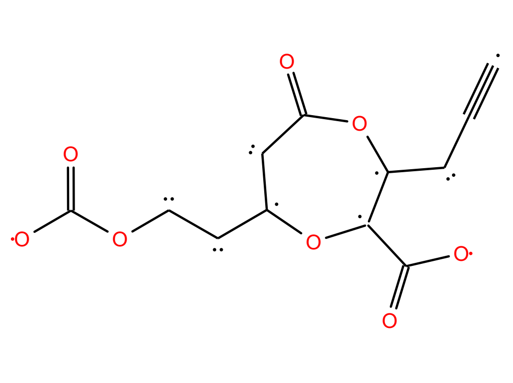

entry name spike_inpaint_ca_mol.40

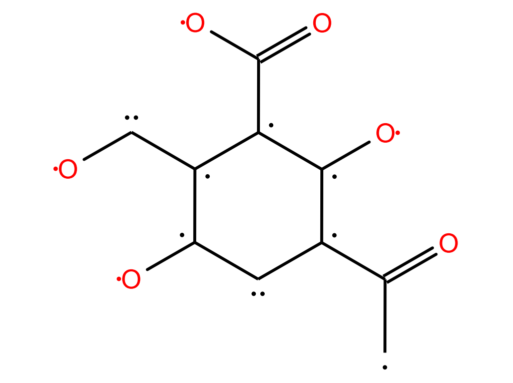

entry name spike_inpaint_ca_mol.41

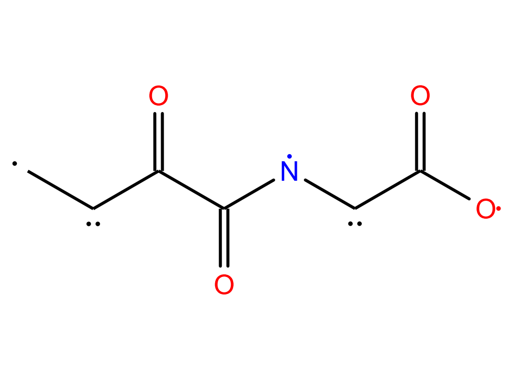

entry name spike_inpaint_ca_mol.42

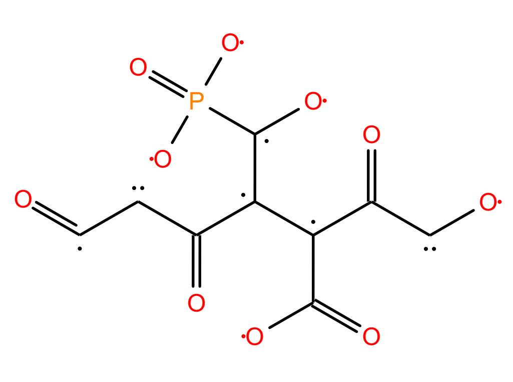

entry name spike_inpaint_ca_mol.43

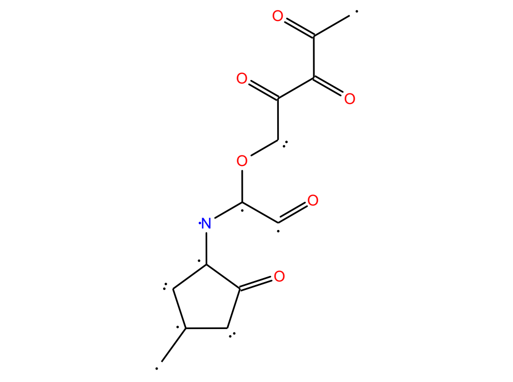

entry name spike_inpaint_ca_mol.44

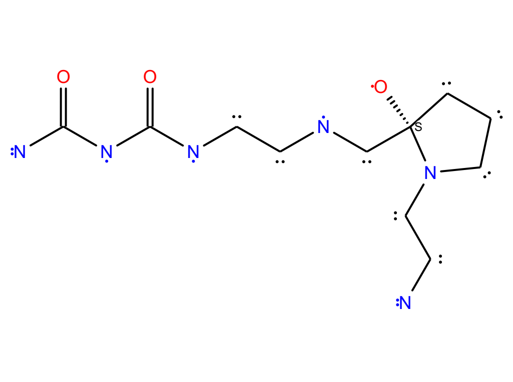

entry name spike_inpaint_ca_mol.45

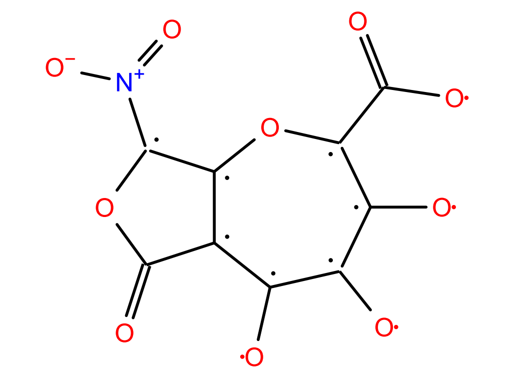

entry name spike_inpaint_ca_mol.46

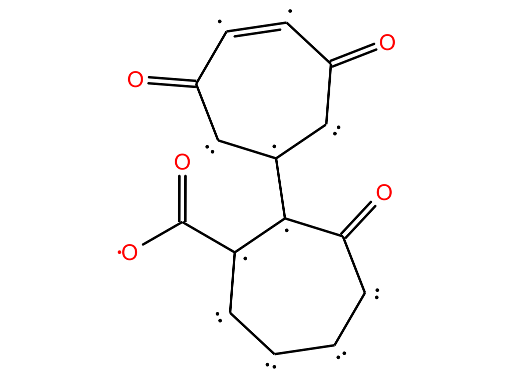

entry name spike_inpaint_ca_mol.47

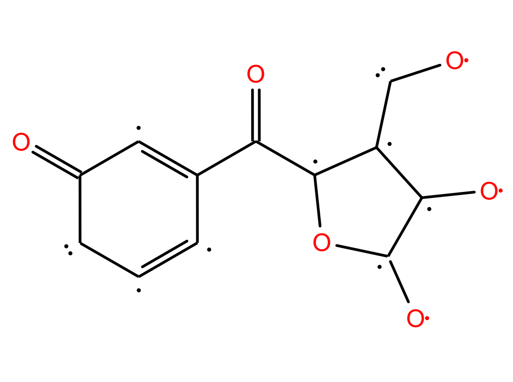

entry name spike_inpaint_ca_mol.48

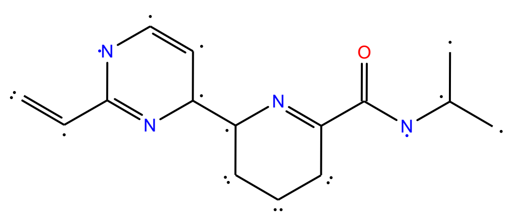

entry name spike_inpaint_ca_mol.49

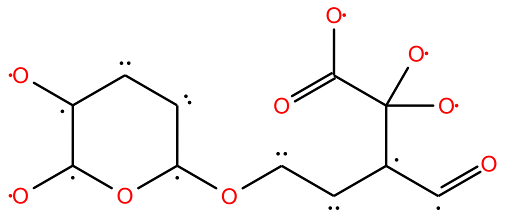

entry name spike_inpaint_ca_mol.50

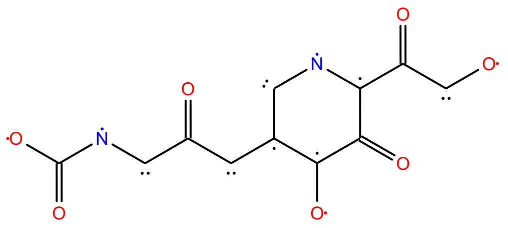

entry name spike_inpaint_ca_mol.51

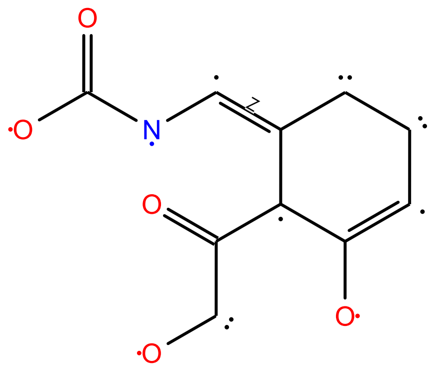

entry name spike_inpaint_ca_mol.52

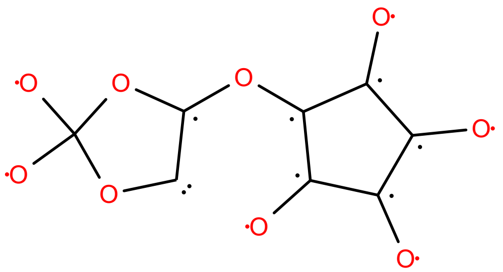

entry name spike_inpaint_ca_mol.53

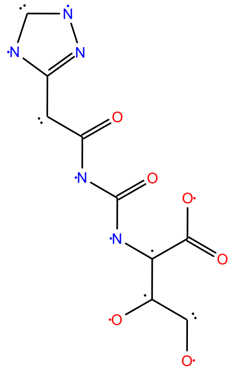

entry name spike_inpaint_ca_mol.54

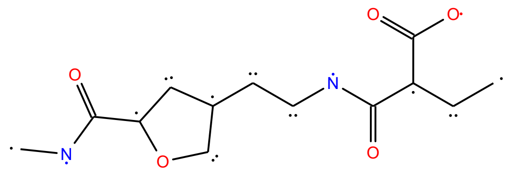

entry name spike_inpaint_ca_mol.55

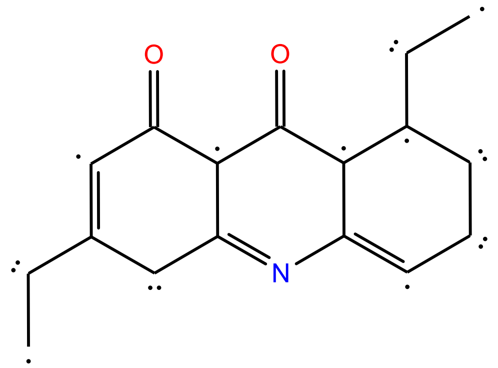

entry name spike_inpaint_ca_mol.56

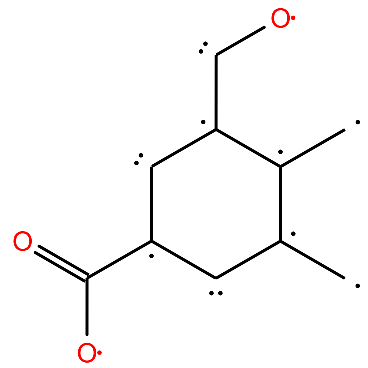

entry name spike_inpaint_ca_mol.57

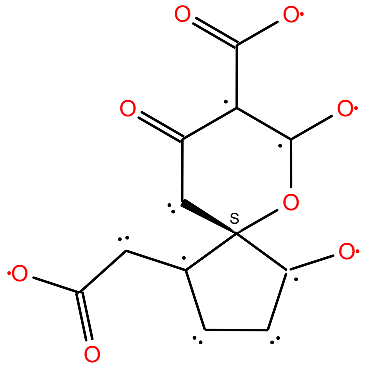

entry name spike_inpaint_ca_mol.58

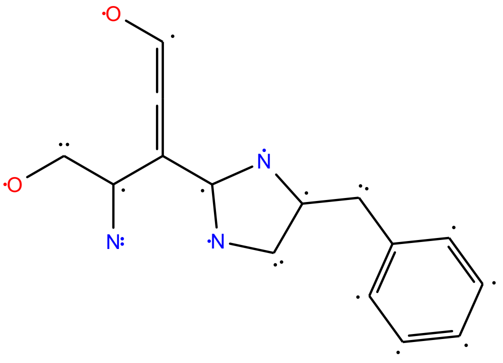

entry name spike_inpaint_ca_mol.59

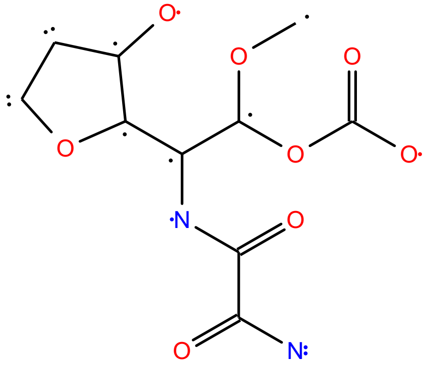

entry name spike_inpaint_ca_mol.60

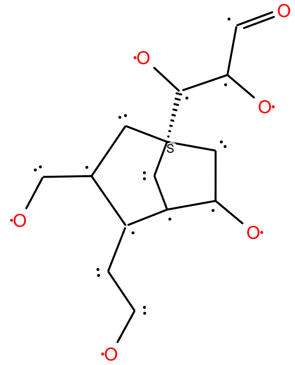

entry name spike_inpaint_ca_mol.61

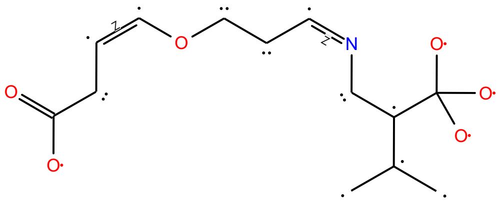

entry name spike_inpaint_ca_mol.62

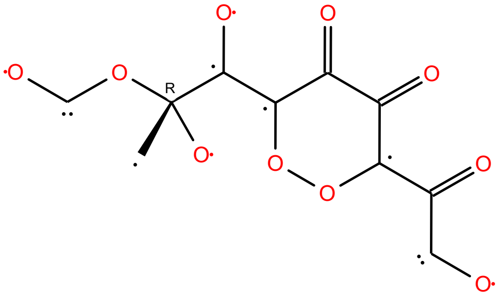

entry name spike_inpaint_ca_mol.63

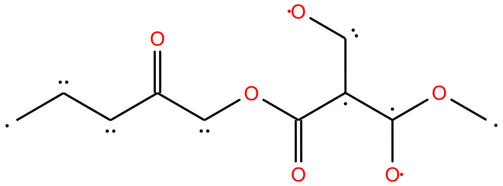

entry name spike_inpaint_ca_mol.64

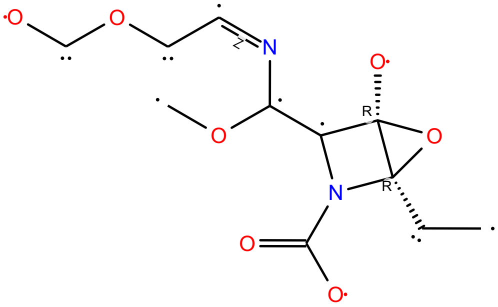

entry name spike_inpaint_ca_mol.65

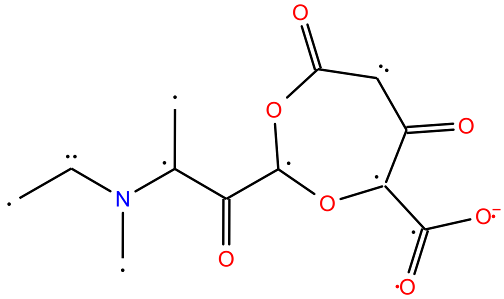

entry name spike_inpaint_ca_mol.66

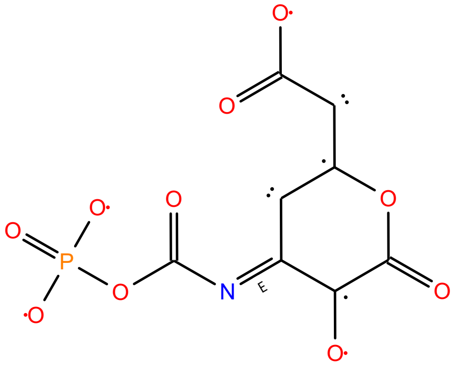

entry name spike_inpaint_ca_mol.67

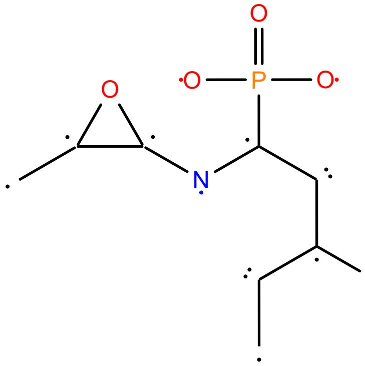

entry name spike_inpaint_ca_mol.68

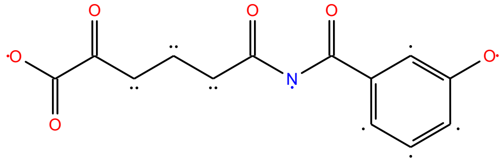

entry name spike_inpaint_ca_mol.69

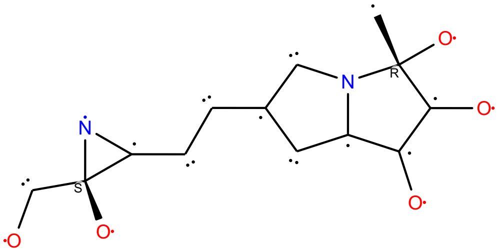

entry name spike_inpaint_ca_mol.70

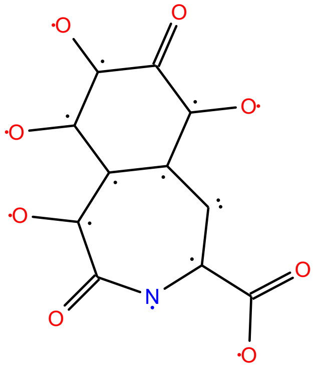

entry name spike_inpaint_ca_mol.71

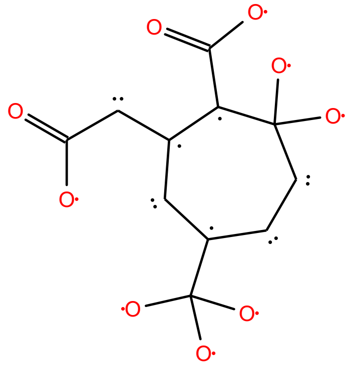

entry name spike_inpaint_ca_mol.72

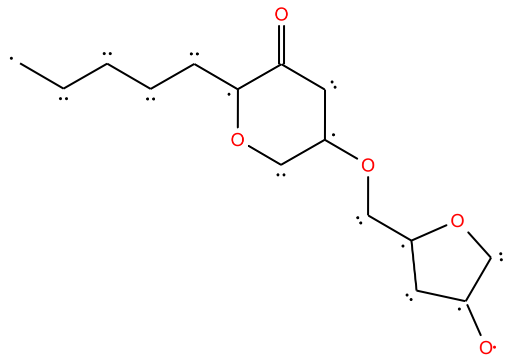

entry name spike_inpaint_ca_mol.73

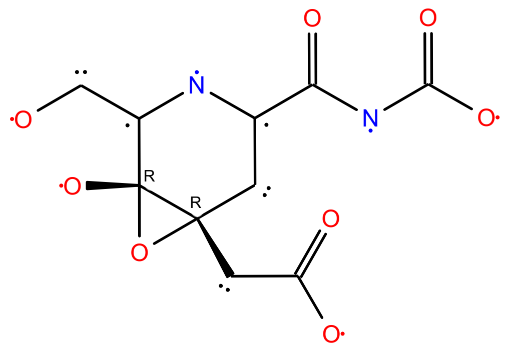

entry name spike_inpaint_ca_mol.74

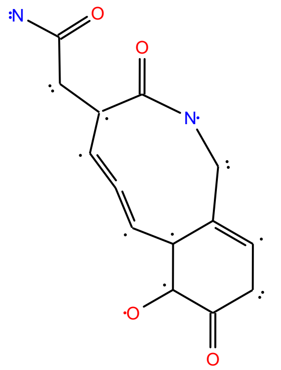

entry name spike_inpaint_ca_mol.75

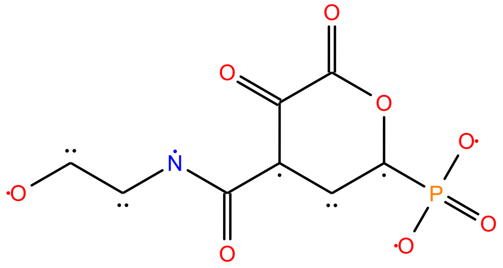

entry name spike_inpaint_ca_mol.76

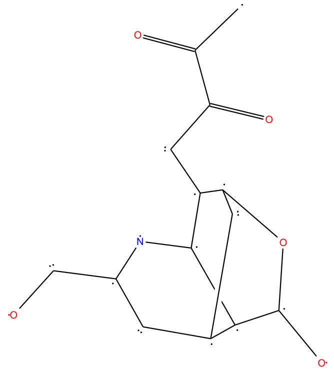

entry name spike_inpaint_ca_mol.77

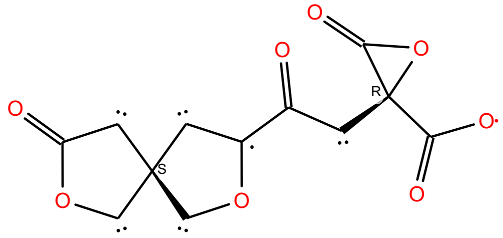

entry name spike_inpaint_ca_mol.78

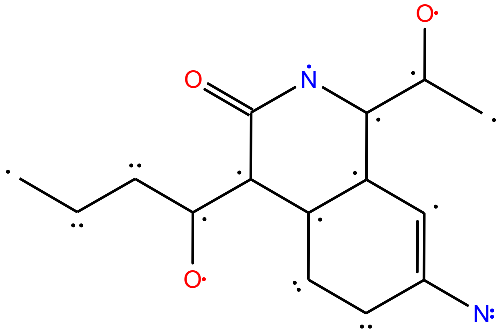

entry name spike_inpaint_ca_mol.79

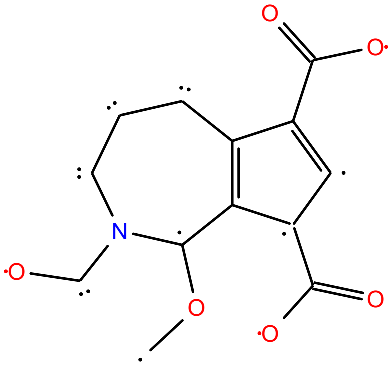

entry name spike_inpaint_ca_mol.80

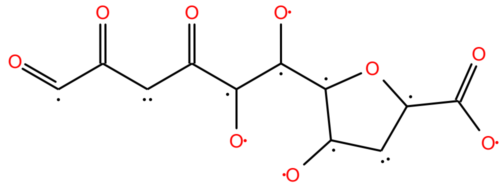

entry name spike_inpaint_ca_mol.81

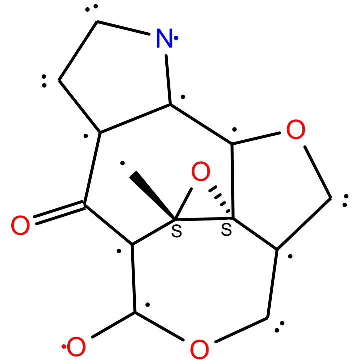

entry name spike_inpaint_ca_mol.82

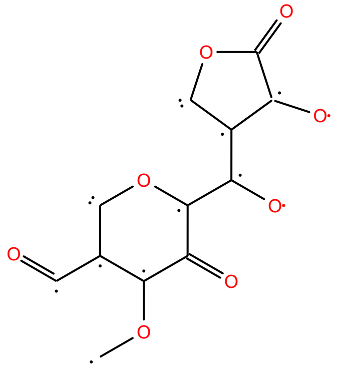

entry name spike_inpaint_ca_mol.83

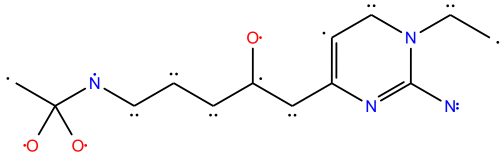

entry name spike_inpaint_ca_mol.84

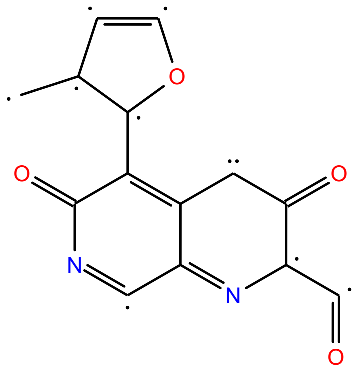

entry name spike_inpaint_ca_mol.85

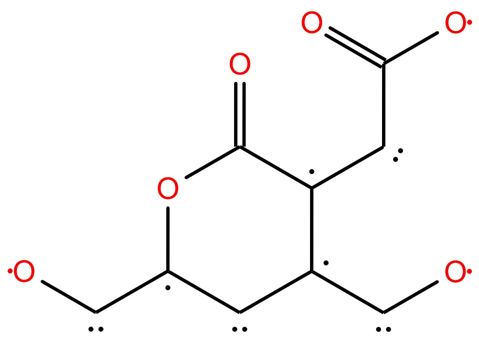

entry name spike_inpaint_ca_mol.86

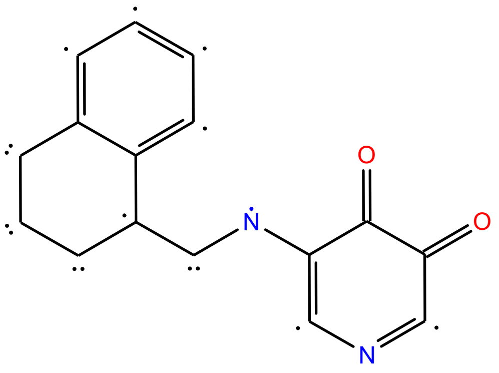

entry name spike_inpaint_ca_mol.87

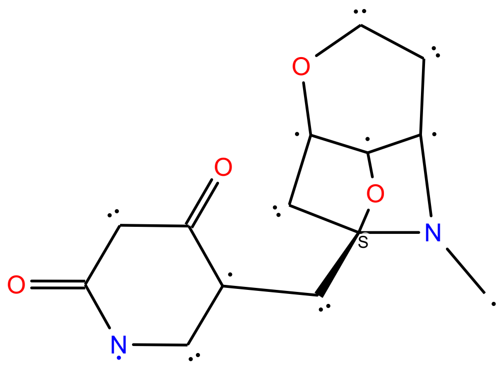

entry name spike_inpaint_ca_mol.88

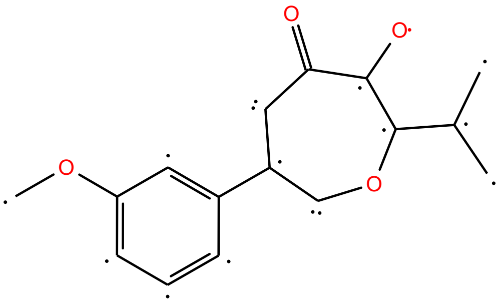

entry name spike_inpaint_ca_mol.89

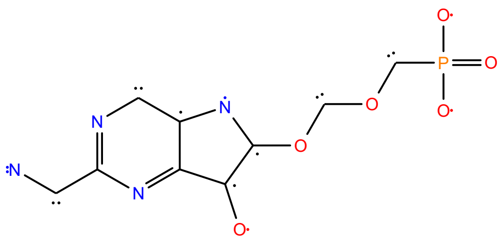

entry name spike_inpaint_ca_mol.90

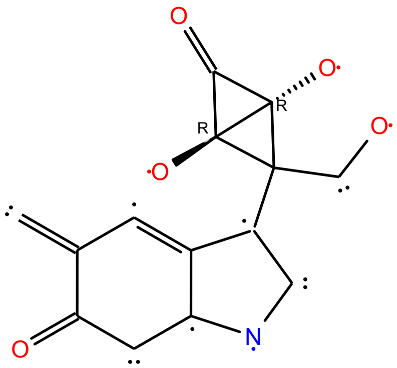

entry name spike_inpaint_ca_mol.91

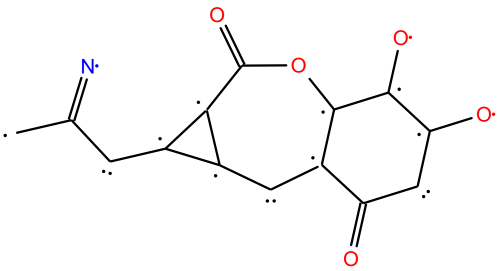

entry name spike_inpaint_ca_mol.92

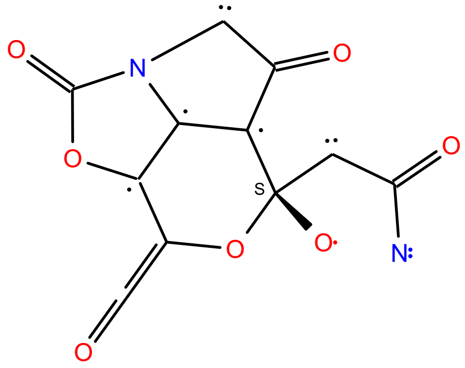

entry name spike_inpaint_ca_mol.93

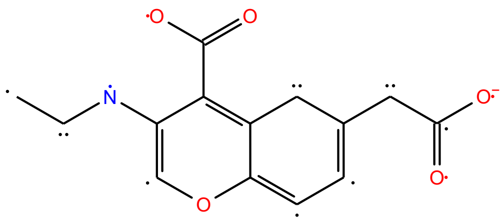

entry name spike_inpaint_ca_mol.94

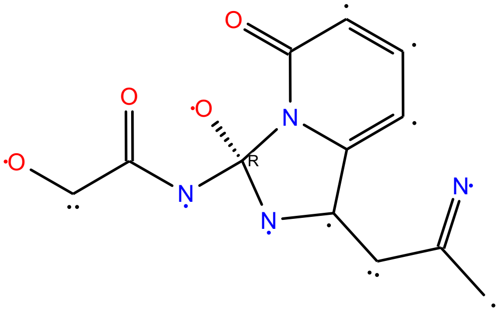

entry name spike_inpaint_ca_mol.95

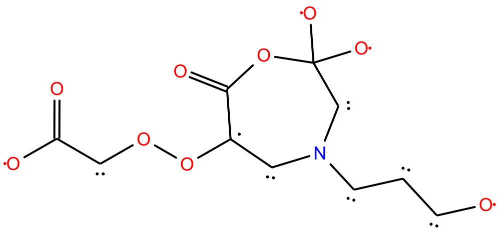

entry name spike_inpaint_ca_mol.96

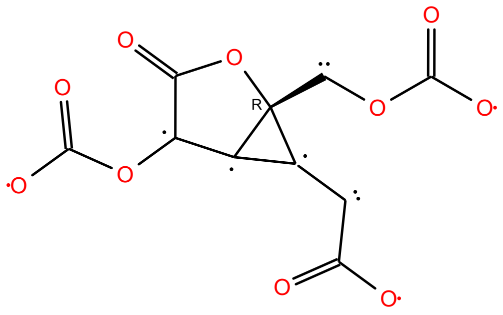

entry name spike_inpaint_ca_mol.97

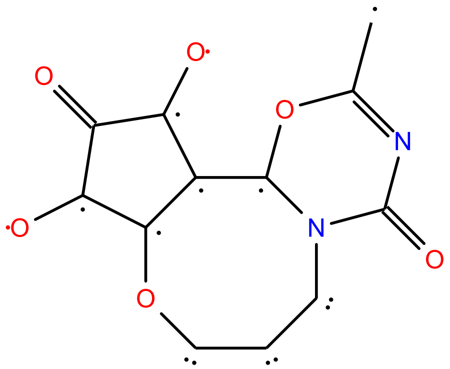

entry name spike_inpaint_ca_mol.98
