# Supplementary material for: De Novo Design of Anti-COVID Drugs Using Machine Learning-Based Equivariant Diffusion Model Targeting the Spike Protein
Source: Curr Issues Mol Biol. 2023 May 12;45(5):4261–84. doi: 10.3390/cimb45050271 (PMC10217495; doi:10.3390/cimb45050271)
Supplement: Supplementary file 1 [file cimb-45-00271-s001.zip › Supplementary files/Supplementary file S4.pdf]

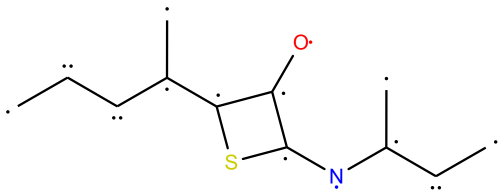

entry name spike_fullatom_new2_m

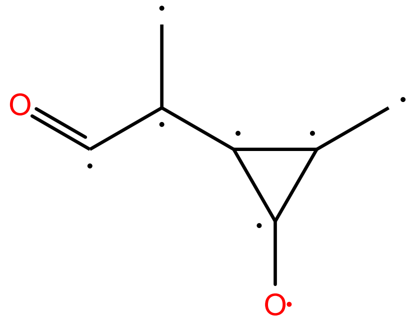

entry name spike_fullatom_new2_m

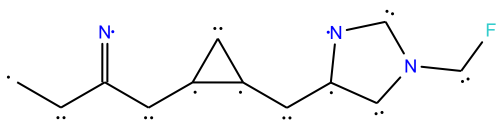

entry name spike_fullatom_new2_m

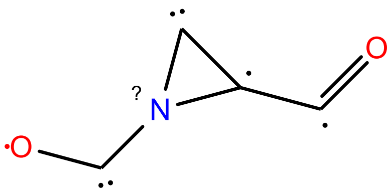

entry name spike_fullatom_new2_m

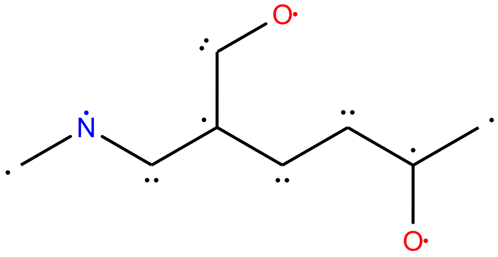

entry name spike_fullatom_new2_m

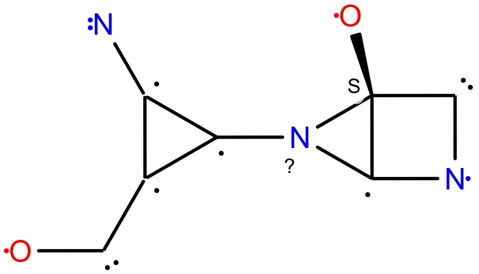

entry name spike_fullatom_new2_m

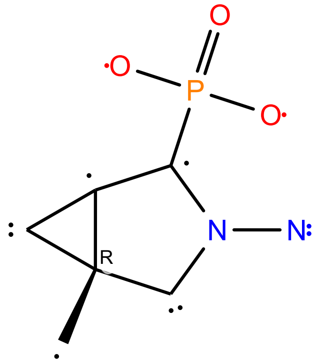

entry name spike_fullatom_new2_m

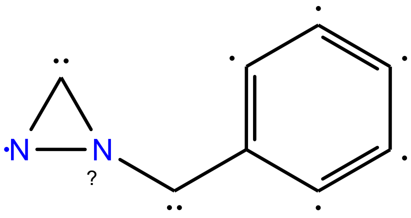

entry name spike_fullatom_new2_m

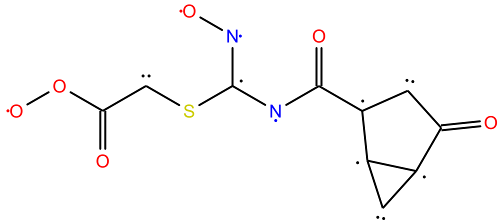

entry name spike_fullatom_new2_m

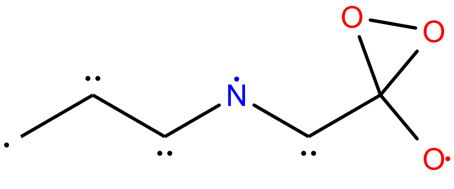

entry name spike_fullatom_new2_m

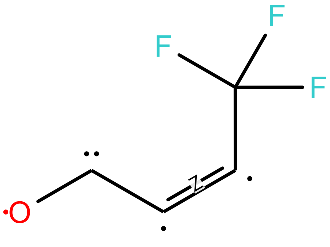

entry name spike_fullatom_new2_m

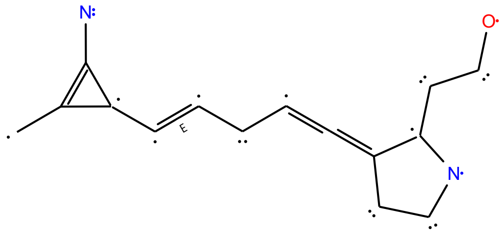

entry name spike_fullatom_new2_m

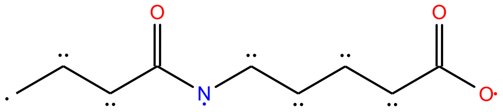

entry name spike_fullatom_new2_m

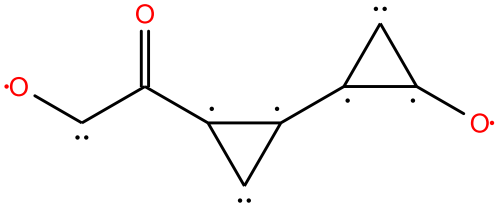

entry name spike_fullatom_new2_m

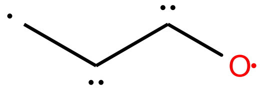

entry name spike_fullatom_new2_m

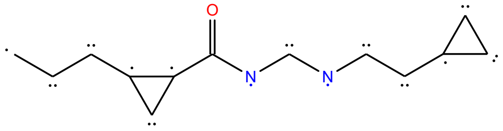

entry name spike_fullatom_new2_m

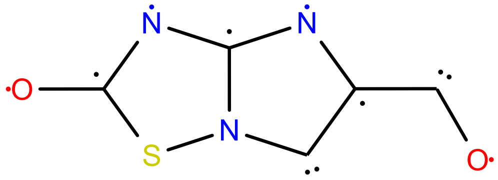

entry name spike_fullatom_new2_m

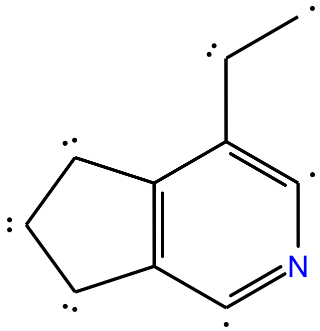

entry name spike_fullatom_new2_m

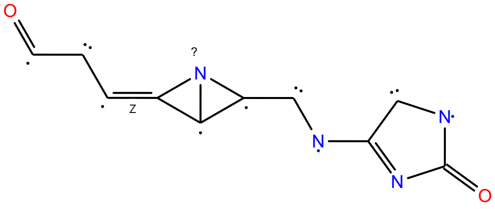

entry name spike_fullatom_new2_m

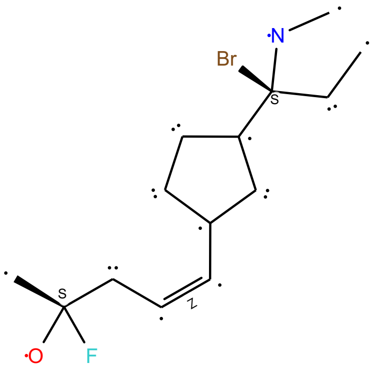

entry name spike_fullatom_new2_m

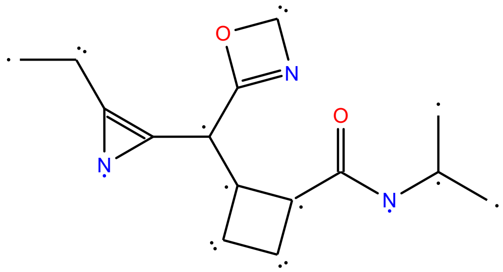

entry name spike_fullatom_new2_m

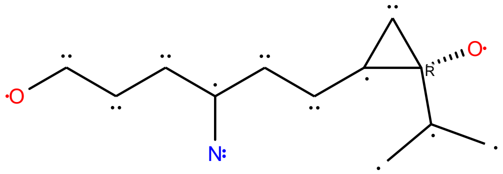

entry name spike_fullatom_new2_m

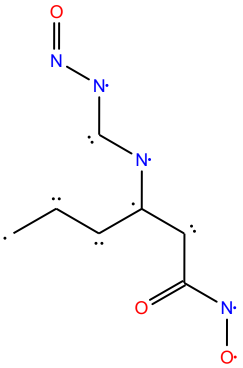

entry name spike_fullatom_new2_m

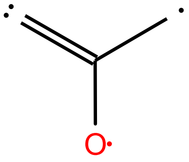

entry name spike_fullatom_new2_m

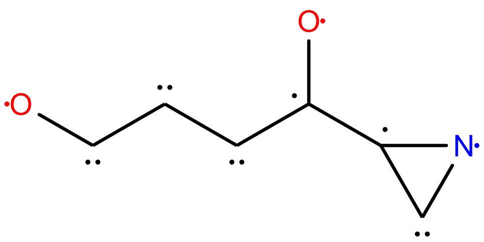

entry name spike_fullatom_new2_m

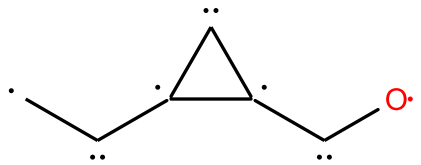

entry name spike_fullatom_new2_m

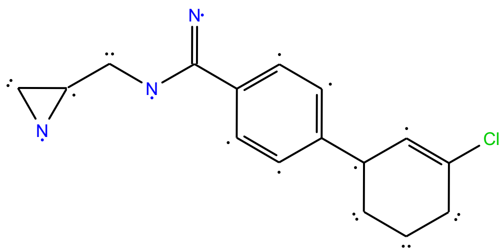

entry name spike_fullatom_new2_m

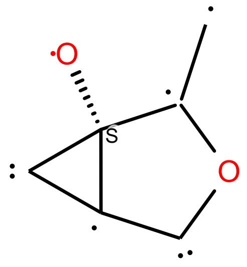

entry name spike_fullatom_new2_m

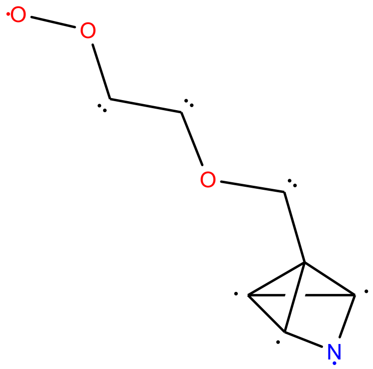

entry name spike_fullatom_new2_m

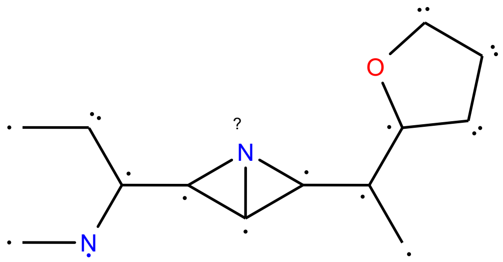

entry name spike_fullatom_new2_m

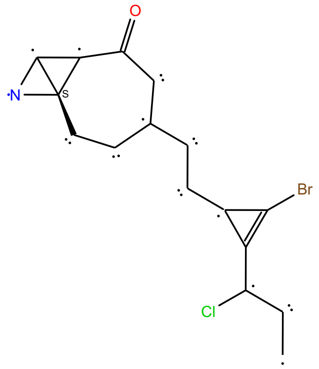

entry name spike_fullatom_new2_m

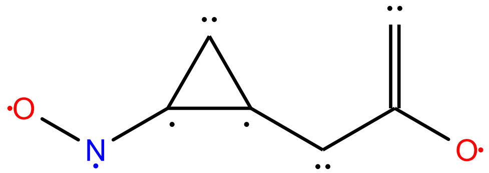

entry name spike_fullatom_new2_m

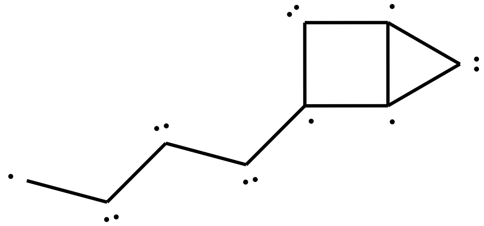

entry name spike_fullatom_new2_m

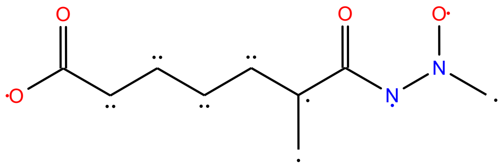

entry name spike_fullatom_new2_m

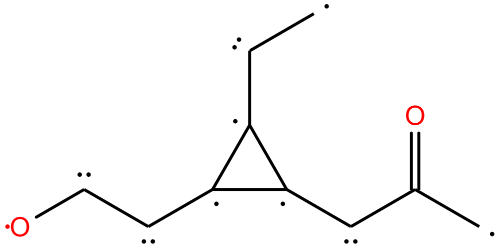

entry name spike_fullatom_new2_m

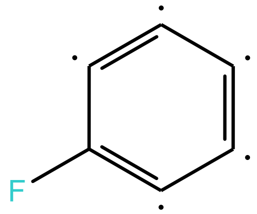

entry name spike_fullatom_new2_m

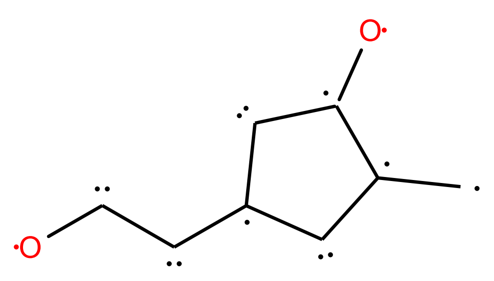

entry name spike_fullatom_new2_m

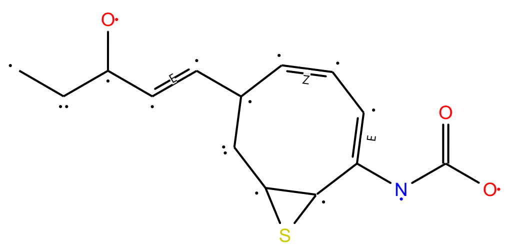

entry name spike_fullatom_new2_m

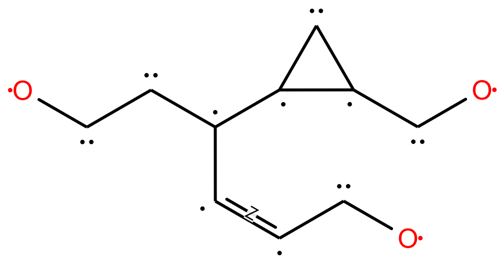

entry name spike_fullatom_new2_m

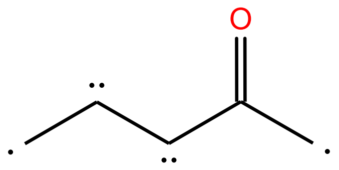

entry name spike_fullatom_new2_m

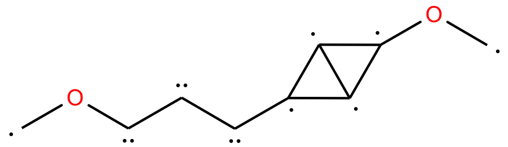

entry name spike_fullatom_new2_m

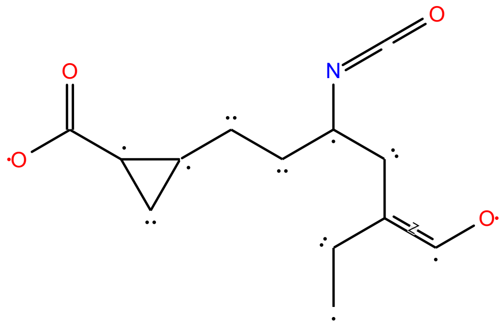

entry name spike_fullatom_new2_m

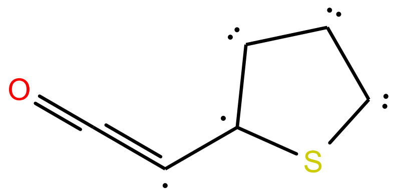

entry name spike_fullatom_new2_m

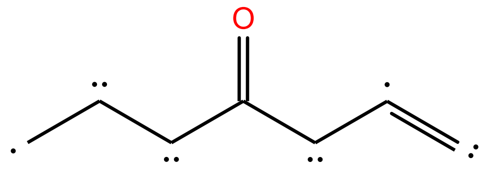

entry name spike_fullatom_new2_m

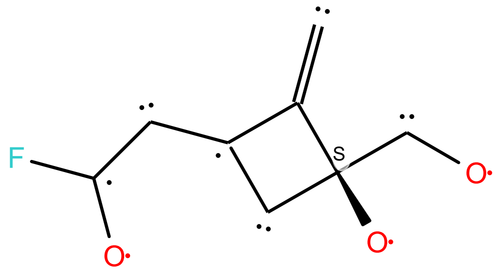

entry name spike_fullatom_new2_m

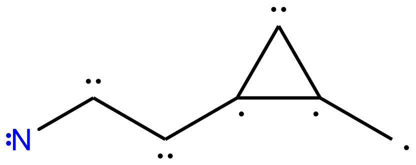

entry name spike_fullatom_new2_m

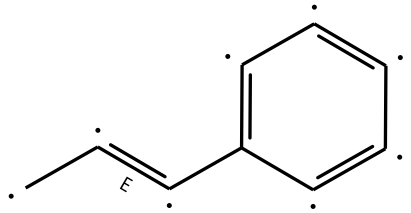

entry name spike_fullatom_new2_m

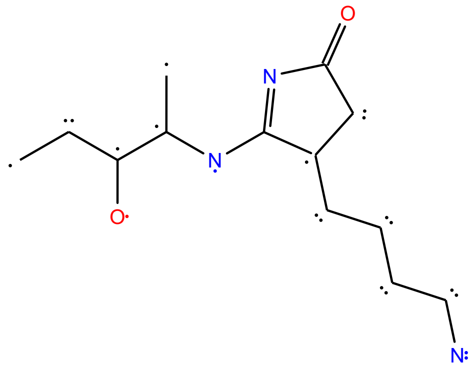

entry name spike_fullatom_new2_m

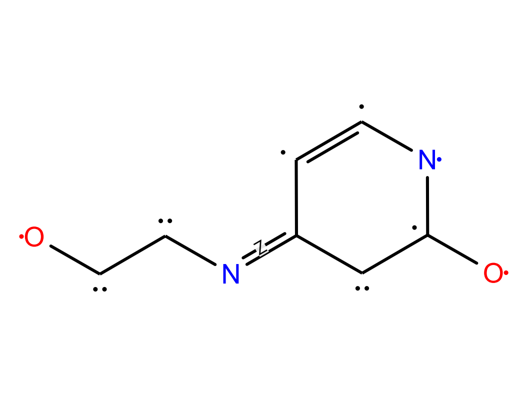

entry name spike_fullatom_new2_m

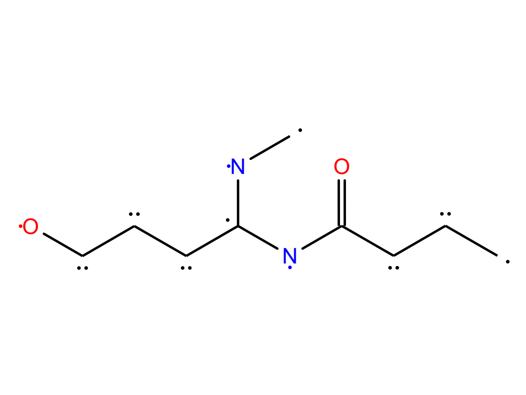

entry name spike_fullatom_new2_m

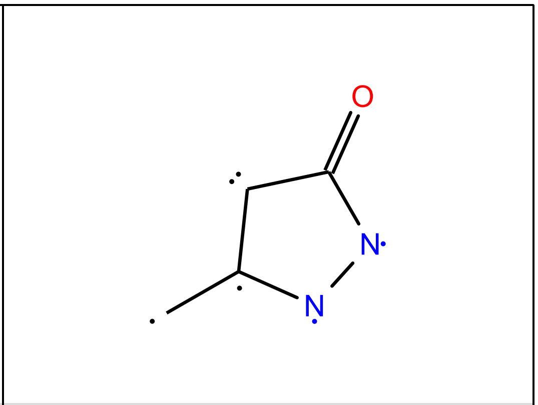

entry name spike_fullatom_new2_m

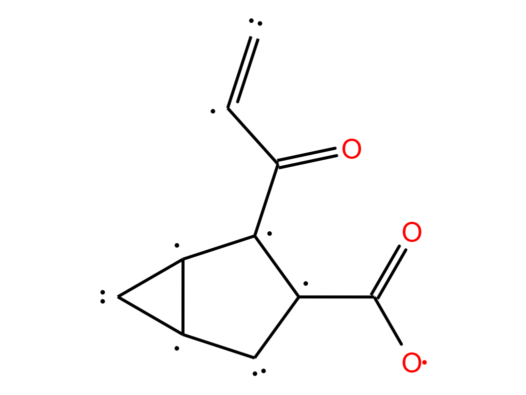

entry name spike_fullatom_new2_m

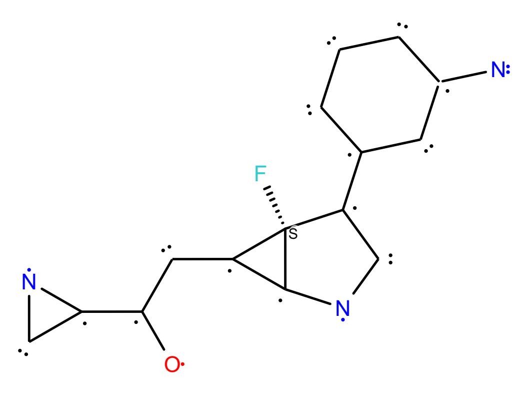

entry name spike_fullatom_new2_m

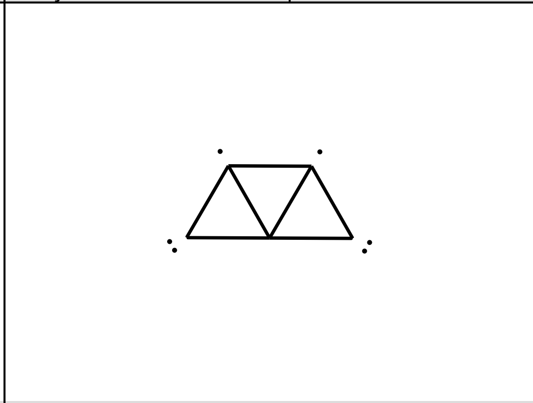

entry name spike_fullatom_new2_m

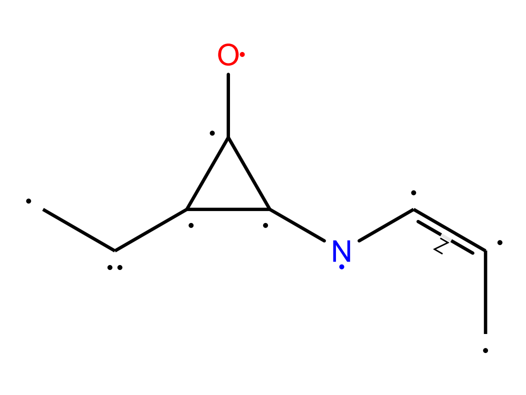

entry name spike_fullatom_new2_m

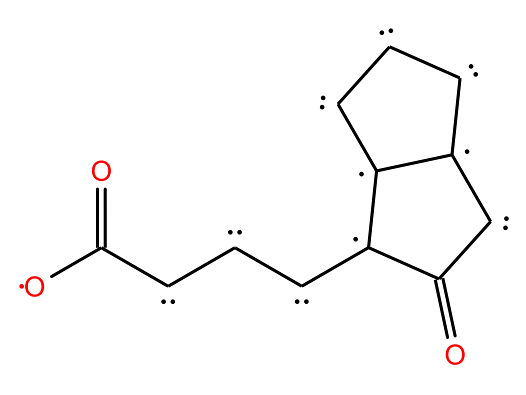

entry name spike_fullatom_new2_m

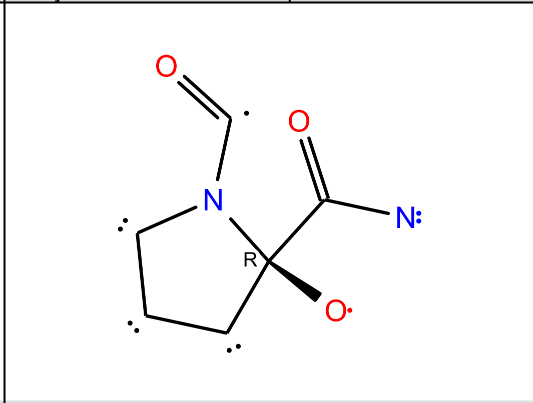

entry name spike_fullatom_new2_m

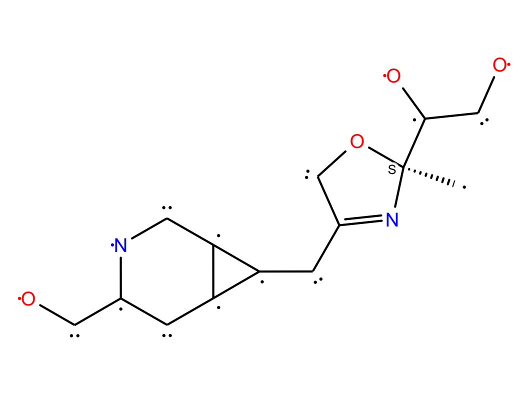

entry name spike_fullatom_new2_m

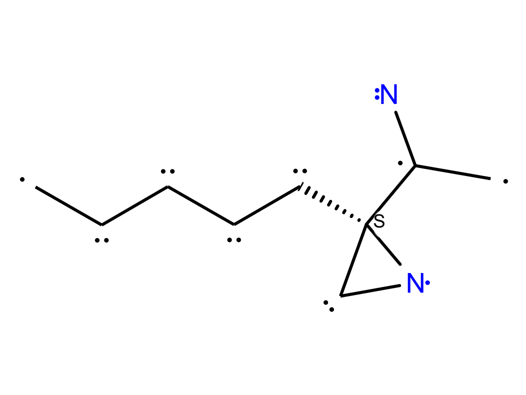

entry name spike_fullatom_new2_m

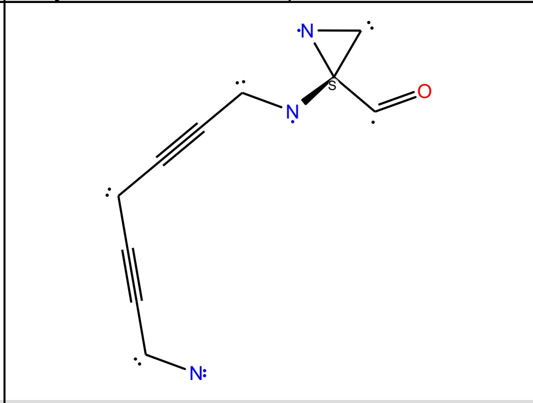

entry name spike_fullatom_new2_m

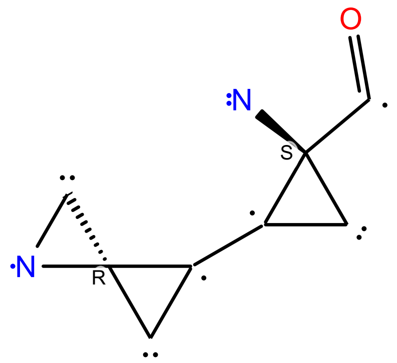

entry name spike_fullatom_new2_m

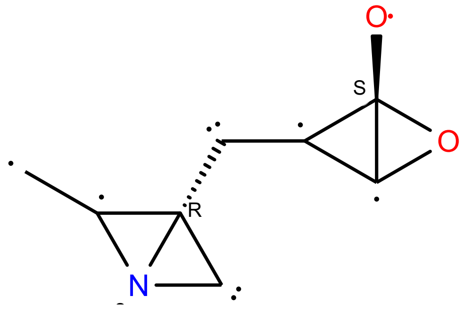

entry name spike_fullatom_new2_m

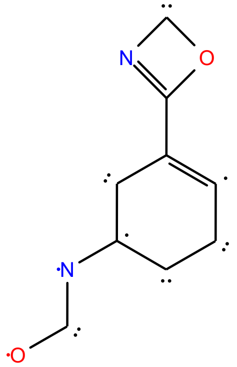

entry name spike_fullatom_new2_m

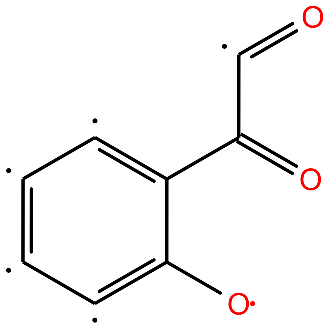

entry name spike_fullatom_new2_m

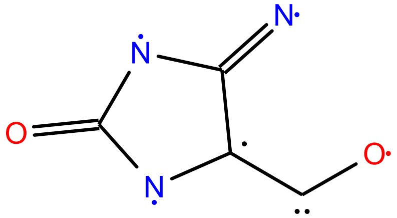

entry name spike_fullatom_new2_m

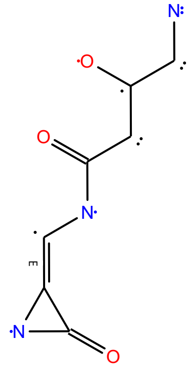

entry name spike_fullatom_new2_m

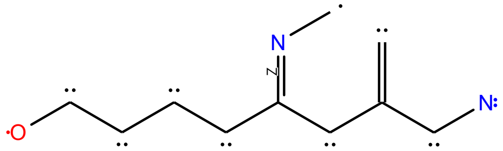

entry name spike_fullatom_new2_m

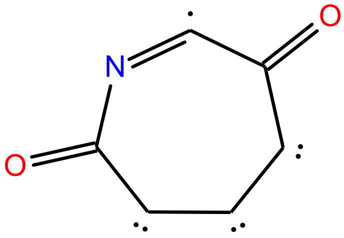

entry name spike_fullatom_new2_m

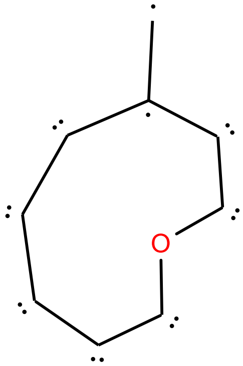

entry name spike_fullatom_new2_m

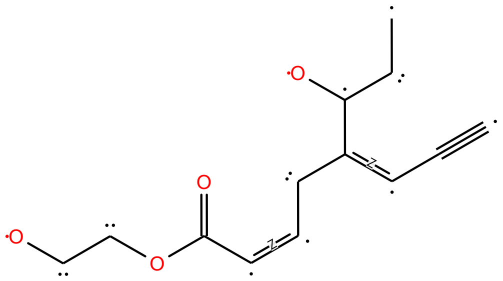

entry name spike_fullatom_new2_m

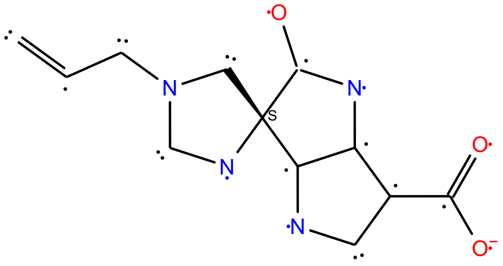

entry name spike_fullatom_new2_m

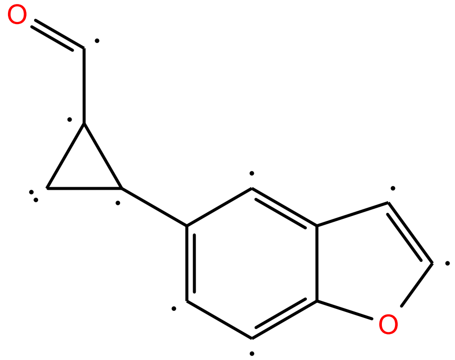

entry name spike_fullatom_new2_m

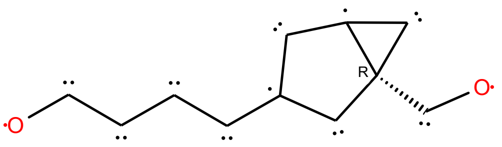

entry name spike_fullatom_new2_m

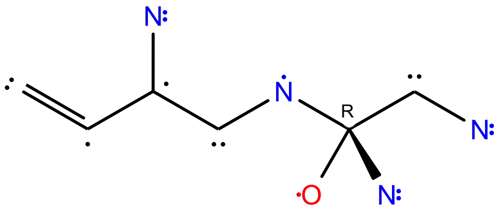

entry name spike_fullatom_new2_m

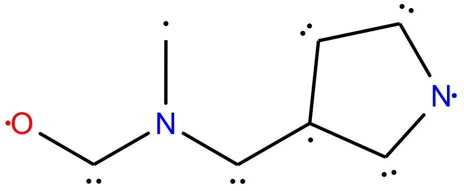

entry name spike_fullatom_new2_m

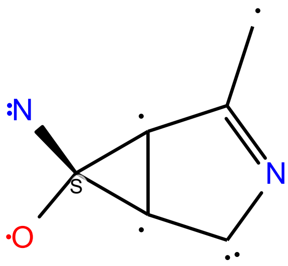

entry name spike_fullatom_new2_m

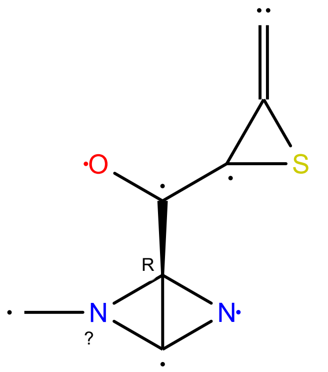

entry name spike_fullatom_new2_m

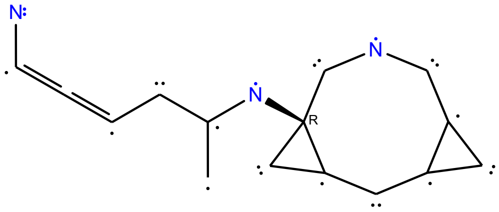

entry name spike_fullatom_new2_m

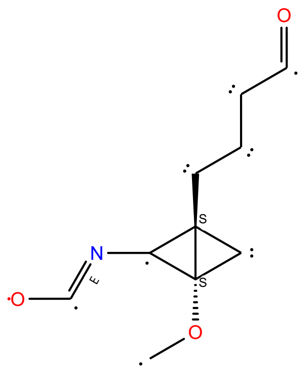

entry name spike_fullatom_new2_m

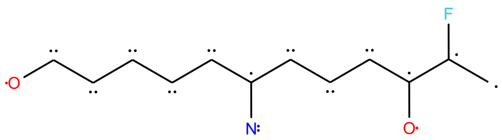

entry name spike_fullatom_new2_m

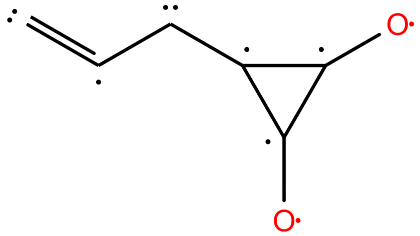

entry name spike_fullatom_new2_m

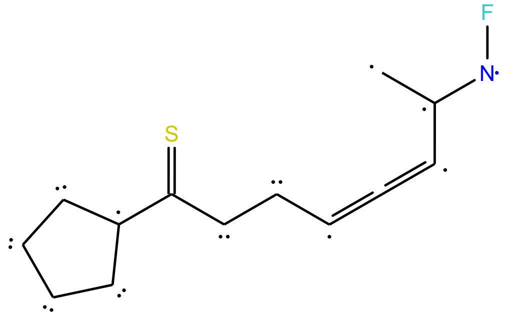

entry name spike_fullatom_new2_m

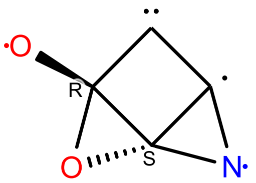

entry name spike_fullatom_new2_m

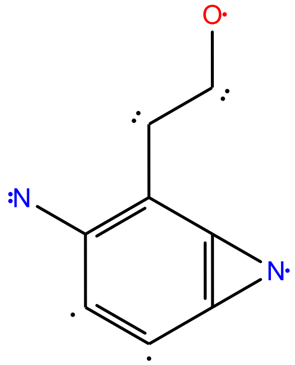

entry name spike_fullatom_new2_m

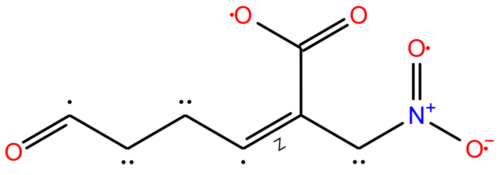

entry name spike_fullatom_new2_m

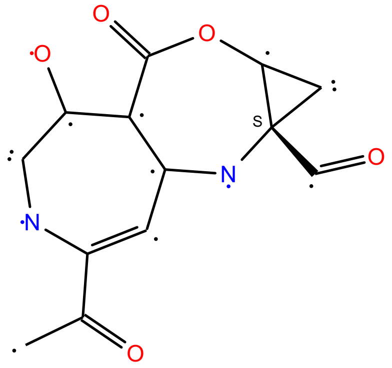

entry name spike_fullatom_new2_m

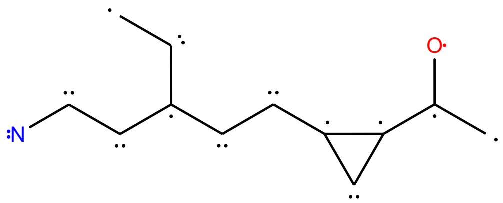

entry name spike_fullatom_new2_m

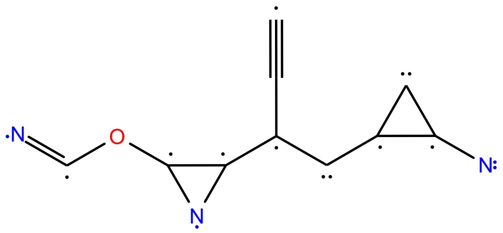

entry name spike_fullatom_new2_m

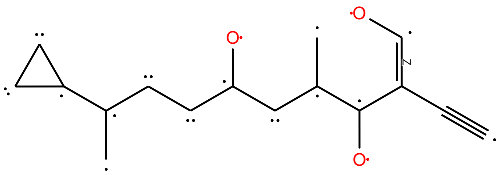

entry name spike_fullatom_new2_m

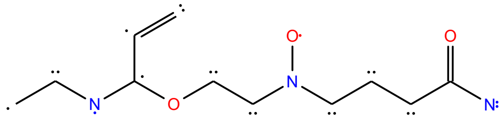

entry name spike_fullatom_new2_m

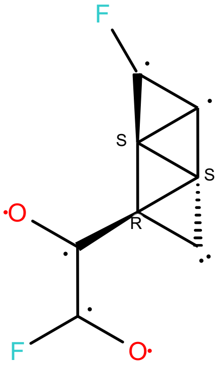

entry name spike_fullatom_new2_m

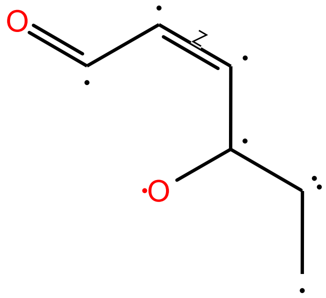

entry name spike_fullatom_new2_m

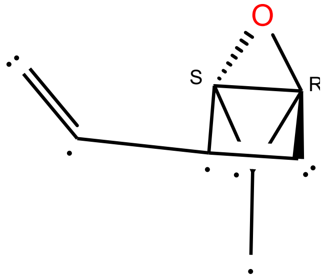

entry name spike_fullatom_new2_m

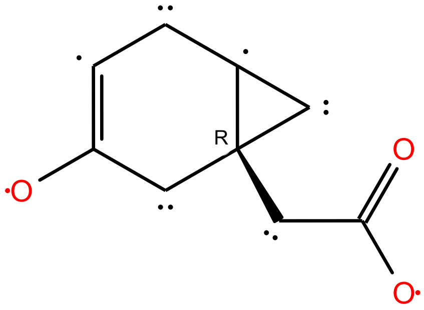

entry name spike_fullatom_new2_m

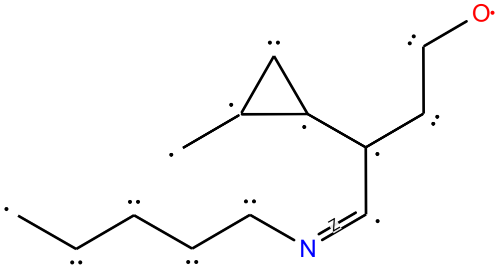

entry name spike_fullatom_new2_m

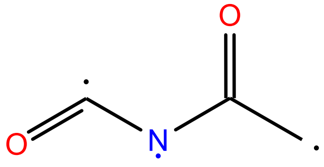

entry name spike_fullatom_new2_m

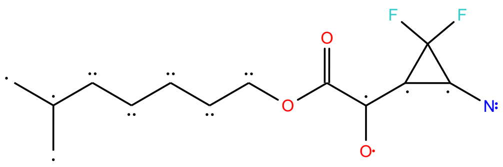

entry name	spike_fullatom_new2_m

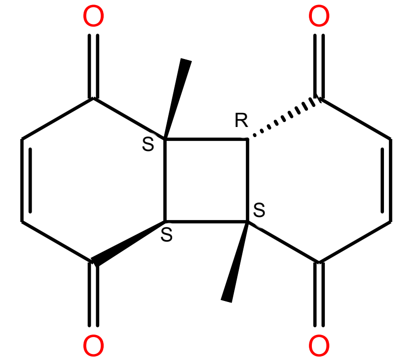

entry name	spike_fullatom_new2_m
